# Supplementary material for: Are carbon emissions trading and green financial instruments synergistic? -Comprehensive quantitative research based on content analysis
Source: PLoS One. 2024 Mar 7;19(3):e0298601. doi: 10.1371/journal.pone.0298601 (PMC10919601; doi:10.1371/journal.pone.0298601)
Supplement: S1 Data — (ZIP) [file pone.0298601.s001.zip › Data/地级市Z_F工作报告.zip!/║ú─╧/║ú┐┌╩╨/└┤╫╘╣1⁄2╓┌║┼í╛┬φ┐╦╩2╛▌═°í┐_2014.docx]

各位代表：

　　现在，我代表市人民政府向大会报告工作，请予审议，并请各位政协委员和其他列席人员提出意见。

　　一、2013年工作回顾

　　过去一年，我们在省委、省政府和市委的正确领导下，以学习贯彻党的十八大、十八届二中、三中全会和习近平总书记一系列重要讲话精神为动力，积极应对各种困难和挑战，着力稳增长、调结构、促改革、惠民生，完成了市十五届人民代表大会第三次会议确定的目标任务。实现全市生产总值904.64亿元，增长9.9%；全社会固定资产投资649.33亿元，增长27.2%；来自海口的公共财政收入240.82亿元，增长14.4%，其中地方公共财政预算收入86.73亿元，增长15.1%；社会消费品零售总额490.05亿元，增长12.3 %；城镇居民人均可支配收入24461元，农民人均纯收入9155元，分别增长9.5%、12.6%；居民消费价格涨幅2.9%；城镇登记失业率1.32%。

　　（一）项目建设取得显著成效

　　深入推进“项目建设年” 。落实“六个一”责任模式，扎实开展“重点项目推进月”、“工业企业调研月”、“园区建设调研月”活动，全年竣工项目60个。90个省市重点项目完成投资412.4亿元，省重点项目达到省政府上台阶奖考核标准。马村港二期中心港区开港运营，新东大桥、海榆中线拓宽等项目基本完工；冯小刚电影公社一期、东寨港红树林旅游风景区一期等景点对外开放；海秀快速路、永秀永和安居工程、司马坡文体基地等项目有序推进；西环高铁海口段全面完成征地拆迁，南渡江引水工程获国家发改委立项批复。

　　积极开展项目招商。与绿地集团、恒大集团、保利地产等21家企业签订合作协议，总投资超千亿元。国务院出台加快棚户区改造决定后，我市创新 “政府让利、企业微利、百姓得利”的投融资模式，优化资源配置，大规模启动灵山镇、五源河、椰岛片区等新一轮棚户区（城中村）改造。这些招商项目不仅实现了投资、消费“双增长”，也为下一步结构调整、转型升级打下基础。

　　（二）产业转型升级取得积极进展

　　三次产业结构比由2012年的6.8：24.6：68.6调整为6.5：24.0：69.5。工业经济稳中提质，在工业总产值与上年持平的情况下，工业增加值同比增长6%。医药产业保持强劲增长，总产值增长11.6%，成为又一个突破百亿的产业集群。美安科技新城完成征地7.5平方公里，一期基础设施加快推进；罗牛山农产品加工产业园一期主体工程基本完工。海马汽车两款新车型投放上市，椰树8万吨椰汁等25个工业项目竣工投产。国家级广告产业园落户高新区，新增23家高新技术企业，我市再次获得“全国科技进步先进市”称号。

　　服务业提质升级步伐加快，第三产业增加值增长10.5%。海口联合农商银行、浦发银行海口分行开业运营，新设小额贷款公司8家，金融业增加值同比增长8.2%。大印橡胶物流保税仓封顶，国家级水产物流交易中心、丁村现代美居、美安物流园建设进展顺利。海口港集装箱吞吐量达到117万标箱、增长16.8%，交通运输业增加值增长17.7%。丰富琼北旅游线路和产品，开通海口至哈尔滨客运列车、海口至西沙邮轮旅游航线，旅游综合效益明显提高，全年接待旅游过夜人数1044万人次，实现旅游收入120亿元，分别增长9.6%、18.3%。美兰机场旅客吞吐量达到1194万人次，同比增长11.6%。成功举办全国图书交易博览会、中国国际物流节、世界基因产业大会、观澜湖高尔夫世界第一挑战赛等节会赛事，全年上规模商务会展活动达到223场次，组织各类岛外促销55场次。房地产业结构调整初见成效，销售商品房338万平方米，同比增长27%。

　　（三）统筹城乡发展取得新成绩

　　积极推进国民经济和社会发展规划、城市总体规划、土地利用总体规划、环境保护总体规划“四规”协调衔接，完成主城区27个片区控规编制，开展9个专项规划和示范镇镇域规划编制，其中城市综合防灾规划、快速路网规划和云龙镇、演丰镇、红旗镇总规控规已经完成。

　　“三农”工作不断加强。农业完成增加值58.5亿元，增长6.3%。落实强农惠农富农补贴资金7966万元。马坡洋蔬菜产业园、新坡兰花产业园核心区一期全面完工。启动罗牛山10万头生猪养殖基地异地技改项目，新增1个无公害农产品基地、15个无公害农产品认证，“红明红”荔枝、三江“豪富江”莲雾被评为省著名商标。积极开展“阳光培训”和“我为农民增收办实事”活动。加大农村基础设施投入，开工建设南渡江流域土地整治15万亩，防洪堤及南面沟泵站加快建设；完成12座小Ⅱ型水库除险加固；新建农村安全饮水工程26宗，解决3.8万人饮水安全问题；完成县道砂土路改造37公里；新建户用沼气池600个；新建村邮站107个；提前完成“十二五”广播电视村村通工程。

　　统筹城乡综合配套改革试点扎实推进。搭建新型城镇化建设运营载体，组建市城乡统筹发展集团。启动云龙、演丰首批示范镇建设，演丰镇第一批17个项目完成投资9.5亿元，云龙镇10个项目正在有序推进，全市百个统筹城乡示范项目全面启动，创新探索具有海口特色的新型城镇化路子。

　　（四）改革开放力度进一步加大

　　继续深化行政审批制度改革，实施审批目录管理，市级行政审批服务事项核减率达70%；推行“三集中、两到位”行政审批模式，开展建设项目并联审批、服务事项联动审批和网上审批，审批效率大幅提高。加快城市社区“扁平化、网格化”管理体制改革，完成1148个社区网格划分，开展9个社区网格化管理服务试点，并在滨海、万福两个社区深度试点。清理盘活财政存量资金，实行财政资金账户统筹管理；完成新一轮市区财政体制调整。市属经营性文化单位转企改制基本完成。推进公立医院改革，在3家医院部分科室试行“先看病、后付费”医疗服务模式，促进市级医院与基层卫生机构结成医疗联合体。有效承接口岸管理职能下放。推进食品药品监督管理体制改革。桂林洋农场各项改革措施有序实施。

　　大力推进对外开放。全年实际利用外资5.12亿美元，增长13.1%；外贸出口18.85亿美元，增长4.84%。秀英港通过国家核心口岸能力建设验收，邮轮边境游异地办证政策落地，开辟海口至越南邮轮、货运航线，新增海口至韩国2条国际航线。加强对外交流合作，推动伦敦大学与华南理工大学合作办学项目落户海口观澜湖，新增2个国际友好城市。大力发展总部经济，大印集团总部落户海口，中国华信海口公司完成贸易额113亿元。深化省会经济圈合作，完成省会经济圈产业规划、旅游规划、南渡江流域生态环境保护规划的课题研究，琼北区域旅游合作初见成效。

　　（五）城市精细化管理进一步强化

　　开展“城市管理年”活动。出台分类处置办法，做到“拆违不拆民心”；强化科技支撑，建立起天上航拍、地面巡查、网上监控的“天罗地网”监管体系；动真碰硬，重拳出击，严厉查处失职、渎职、权钱交易的公职人员；加班加点，连续作战，全年拆除各类违法建筑226.8万平方米。深化道路交通专项治理，“11+1”道路贯通工程完工通车，开通、优化公交线路27条，新投放新能源公交车100辆，投入公共自行车3000辆，被列为全国第二批公交都市创建城市。优化滨海大道、国兴大道交通组织，严厉查处交通违法行为，城市道路交通拥堵状况正在缓解。扎实开展“绿化宝岛”大行动，完成造林面积2.6万亩。实施高排放机动车区域限行，启动整治建筑工地、“泥头车”扬尘污染，空气质量在全国74个实施新标准的城市中排名第一。加强水环境保护，采取关停搬迁和异地技改养殖场、退塘还林、生态修复等措施，开展东寨港红树林自然保护区环境综合整治，区域内水质明显改善。启动海甸五西路排涝泵站、白沙门污水处理厂（一期）改造、垃圾焚烧发电厂扩建、长流生活垃圾转运站等工程建设，完成8个镇、88个行政村、614个自然村的农村生活垃圾收运工程，城乡环境卫生面貌不断改善。

　　强化资源管理。采取收回、收取闲置费、督促开发等方式，开展闲置土地清理，全面完成4.49万亩闲置土地处置，规范了土地市场秩序。完善土地征收和土地储备机制，提前谋划、科学安排土地战略储备，保障基础设施和重点项目建设落地。强化森林资源保护，严格征占林地审核审批，依法处置了一批历史遗留问题。

　　狠抓社会治理。开展“命案必破”、“攻坚禁毒”、“打黑除恶”、“铁拳禁赌”等专项整治行动，积极构建立体化治安防控体系，社会治安形势稳定。深入推进“六五”普法。构建“大调解”新机制，化解各类矛盾纠纷10087宗，办理法律援助案件8206件。建立领导干部信访积案化解制度，基本化解红旗糖厂、戎居公寓等160个重点难点信访积案，解决2768宗房产办证遗留问题。食品药品和质量技术监督进一步加强，安全生产形势保持总体稳定。综合治理出生人口性别比，第三次获得“全省人口和计划生育工作先进市”。广泛开展群众性精神文明创建活动，全社会文明程度不断提高。完成第七届村委会换届选举。军民融合发展，国防动员、人民防空、优抚安置扎实推进。统计、口岸、粮食、气象、保密、档案、史志、民族宗教、外事侨务、对台事务等工作取得新成绩，工会、共青团、妇女儿童、老龄、残疾人、慈善等事业取得新进步。

　　（六）人民生活持续改善

　　全年民生支出90.5亿元，同比增长13.5%，占地方一般预算支出的68.6%。省政府10件民生实事和市委、市政府20件民生实事全部兑现。

　　稳定和扩大就业。发放创业小额贷款6101万元，城镇新增就业5.6万人、增长11.2%，城镇登记失业人员再就业1.39万人，农村富余劳动力转移就业1.09万人，“零就业”家庭动态清零。

　　继续完善社会保障体系。企业退休人员月人均养老金提高到1683元，农村“五保”供养月标准提高到480元，城乡低保月标准分别提高到450元和360元，提前完成“十二五”目标；出台《被征地农民参加社会养老保险方案》。新农保参保26.5万人，城镇居民养老保险参保4.5万人，参保率均达97%。提高城镇居民医保、新农合财政补助标准，不断完善城乡医疗救助。开展居家养老服务试点，建成141个社区居家养老服务站；完成4家农村敬老院建设，新增床位540张。

　　加大保障性住房建设。新建保障性住房10453套，全面完成3500户农村危房和88户水库移民危房改造；出台公共租赁住房、限价商品住房《管理暂行办法》，降低保障准入门槛，配售各类保障性住房7435套，配租廉租公租住房1475套，对3358户家庭发放廉租房补贴1085万元。

　　积极平抑物价。在全省率先安排1500万元防控禽流感，扶持养禽业生产，稳定市场物价。建成4个公益性蔬菜批发市场，开设37家农副产品平价商店。提前做好节假日、恶劣天气鲜活农产品的市场储备和供应。安排1.02亿元价格调节基金，对“菜篮子”工程建设、流通领域、低收入群众、在校学生、福利院、敬老院实行补贴。强化价格执法监督，保持物价总水平基本稳定。

　　积极发展社会事业。着力规范办学行为，在全省率先实施城区义务教育阶段学校网上申请学位。合理配置教育资源，整合撤并城区中小学校21所，新建公办幼儿园14所， 301个“中小学校安工程五年规划”项目全部竣工。继续完善农村医疗机构标准化建设，城镇居民规范化电子健康档案建档率达80.2%，免费为城乡居民提供12项基本公共卫生服务和5项重大公共卫生服务。扎实开展文化惠民工程，完成秀英炮台修缮和世纪公园市民文化广场建设，组织精品文艺演出22场、送戏送电影下乡3097场次。

　　各位代表，过去一年，我们按照“干部清正、政府清廉、政治清明”的要求，进一步加强政府自身建设。认真落实中央、省、市关于改进工作作风、密切联系群众的各项规定，“三公”经费同比下降21.8%。加强审计监督，强化政府债务、财政资金和重大项目的跟踪审计。加强民主法制建设，及时向人大常委会报告工作，向政协通报情况，办理人大代表建议363件、政协委员提案370件，依法行政工作实现全省“五连冠”。拓宽网络问政渠道，《问政海口》获得第四届“全国网络问政突出贡献品牌奖”。深入开展“庸懒散奢贪”治理和工程建设、土地利用、城管执法等领域专项治理，纠正部门和行业不正之风，完善惩治和预防腐败体系，人民群众对政府廉政建设的满意度持续提高。

　　各位代表！过去一年的成绩来之不易，是省委、省政府和市委坚强领导的结果，是全市人民团结拼搏的结果。在此，我代表市人民政府，向全市人民致以崇高的敬意！向人大代表、政协委员，向各民主党派、工商联、各人民团体和社会各界人士，向驻市部队、武警官兵和公安司法干警，表示衷心的感谢！

　　在总结成绩的同时，我们也清醒地看到发展中还存在不少困难和问题：一是投资结构不尽合理，第一产业和第二产业投资不足。二是社会消费需求不旺，增幅处于低位。三是实体经济、产业基础相对薄弱，需要多元产业支撑。四是资源环境约束压力加大，社会治理和城市管理面临新的挑战。五是农村基础设施和公共服务配套不足，脱贫任务依然艰巨。六是有的政府工作人员服务意识、办事效率和执行能力还不适应发展需要，整体素质有待提高。对此，我们必须认真对待，妥善解决。

　　二、2014年目标任务

　　2014年是落实党的十八届三中全会精神、全面深化改革的开局之年，也是完成“十二五”规划目标任务的关键之年。做好今年政府工作要全面贯彻落实党的十八大、十八届二中、三中全会、习近平总书记一系列重要讲话精神和省委六届五次全会精神，按照市委十二届六次全会的部署，坚持稳中求进的总基调，以开展党的群众路线教育实践活动为契机，以改革创新、科学管理和作风建设为抓手，着力推动经济提质增效升级，积极稳妥推进新型城镇化，强化和创新社会治理，进一步保障和改善民生，加快转变政府职能，努力实现经济持续健康发展、社会和谐稳定。

　　今年经济社会发展的主要预期目标是：全市生产总值增长10%；地方公共财政预算收入增长15%；全社会固定资产投资增长23%；社会消费品零售总额增长12.5%；城镇居民人均可支配收入和农村居民人均纯收入分别增长10%和11%；居民消费价格涨幅控制在5%以内；城镇登记失业率控制在3%以下。

　　各位代表！建设海南“首善之城”，是省委、省政府对海口的殷切期望；打造“四宜三养”最精最美省会城市，是全市人民共同的责任担当。今年，政府工作将牢牢把握好以下五点：第一，突出稳中求进。海口仍处于欠发达阶段，经济规模小，发展仍是解决一切问题的关键。只有保持一定的发展速度，才能壮大经济规模，为结构调整腾出空间，为提质增效升级创造条件。第二，突出改革创新。按照中央和省里统一部署，在深化改革中强化省会城市的责任担当，把改革创新贯穿于经济社会发展的各个领域各个环节，从社保、教育、医疗等人民群众最期盼的领域改起，从简政放权、行政审批等影响经济社会发展最突出的问题改起，增创体制机制新优势，增添科学发展新活力。第三，突出科学管理。城市越发展，越需要加强管理，管理与发展同等重要。必须下大力气突出抓好规划管理、资源管理、环境管理、交通管理、社区管理等。第四，突出民生优先。尽力而为，量力而行，在就业、社保、住房等民生领域保基本、兜底线、补“短板”，更加关怀弱势群体生活，更加关心农民群众增收，更加注重人民生活环境的改善，让改革发展成果更多更公平地惠及全市人民。第五，突出作风建设。坚持问题导向，不折不扣地贯彻改进工作作风、密切联系群众的各项规定，持之以恒地反对形式主义、官僚主义、享乐主义和奢靡之风，更加及时有效地为基层、企业和群众解决实际问题，切实将党的群众路线教育实践活动的成效转化为惠民利民成果。

　　三、2014年工作重点

　　（一）开展“深化改革年”活动，推进重点领域改革

　　认真贯彻市委《关于推动海口全面深化改革的实施意见》，按照“深化改革年”的各项工作部署，重点推进以下六项改革。

　　深化农村改革。在全市镇域范围内开展农村土地确权登记颁证，完成云龙、演丰、红旗农村土地确权颁证到户。稳妥开展农村集体经营性建设用地和农村集体土地承包经营权流转试点，出台试行办法，逐步建立全市统一的公共资源交易平台体系。探索农村建设用地使用权、承包经营权等产权抵押融资，争取全省农村金融综合改革试点。开展农村集体土地“三过”问题专项清理，加强农村集体留用地管理。完善征地补偿机制，切实维护被征地农民合法权益。完成三江农场整建制移交接收工作。启动城乡一体化户籍制度改革。

　　推进国资国企改革。出台《全面深化国资国企改革的实施意见》，以管资本为主加强国有资产分类监管。对全市国有国资企业进行梳理，选择1-2家市属国有企业进行改革重组和资源整合，引导重点市属国有企业发挥好孵化项目、培育产业、投资重大基础设施和保障基本公共服务的功能。支持港航控股落实海口港与洋浦港重组协议，制定分步走的具体实施方案，完成琼北港口资源的初步整合。改革国有资本授权经营体制，完善企业法人治理结构。强化国有企业经营投资责任追究。

　　加快投融资体制改革。按照国家、省统一部署，及时研究制定负面清单细则。继续推进资源撬动资本，清理整合产业政策体系，通过政策引导、资源配置，鼓励社会资本、非公有制企业投向基础设施、棚户区（城中村）改造、生态环保、公共服务等领域。完善政府投资项目管理模式，修订和出台《政府投资项目管理暂行办法》、《政府投资项目概算包干管理办法》。

　　深化财税体制改革。逐步完善事权与支出责任相匹配的财政体制，增强区级财政统筹能力，确保市区财政体制调整上半年运行到位。清理整合专项转移支付，加大一般性、财力性转移支付力度。全面推进以项目库为基础的部门预算编制改革，试编本级部门补充项目预算，实行预决算、部门预算和“三公”经费公开。完善债务归口管理机制，加强债务风险管控。着力改善办税服务环境，完善新型纳税服务体系。

　　深化教育文化医药卫生体制改革。积极引进国内外知名大学、职业教育、科研机构设立分校分院，推动海口华南伦敦大学获教育部批准并开工建设。创新义务教育管理体制，探索集团化办学模式，扶持发展民办教育。稳步推行校长教师交流轮岗制度，逐步实行城乡义务教育资源均衡配置。积极推进公立医院改革和“先看病、后付费”诊疗服务模式。逐步完善合理分级诊疗模式，推进建立社区医生和居民契约服务关系。健全大病医疗保险和疾病救助制度。推进医师多点执业，鼓励社会办医。

　　加强社区管理体制改革。上半年完成社区网格员配备，年内建成社区扁平化、网格化综合信息平台，实现主城区社区网格管理全覆盖。完善社区医疗、教育、商业等服务设施，选择部分社区开展志愿者服务和市场化服务试点，逐步推进社区管理服务与居民需求无缝对接。加大社区硬件投入，继续改善社区办公和便民服务条件。

　　进一步扩大对外开放。加快推进国家侨务交流示范区基础设施和服务设施建设，办好“世界城市和地方政府联合组织（UCLG）”世界理事年会、亚洲市长论坛和中国外贸500强企业论坛。利用和落实好省市新的航空奖励政策，增加海口至东南亚、港澳台的航线航班。继续推进国际旅游岛各项政策在海口落地。依托综保区，年内建成国际商品（大宗）交易中心和高端商品展示交易中心，探索跨境电子商务和融资租赁业务先行先试。加大柔性引进人才计划推进力度，重点引进高端领军人才。继续推进省会经济圈建设，深化区域旅游、交通、产业布局、医疗服务、环境保护等领域合作。

　　（二）强化多元产业支撑，抓好经济提质增效升级

　　继续抓好投资和项目建设。全年实施省重点项目63个，年度计划投资303亿元。重点加快新型工业升级、商业物流、文化博览、旅游吸引物建设、城市更新改造、新型城镇化、农业现代化、教育医疗完善、生态环境保护、城市基础设施等体现导向性、引领性、辐射性、带动性和示范性的“十大工程”建设，逐一建立市级领导责任制度，确保完成年度项目建设任务。优化投资结构，研究房地产业反哺农业的投入措施，切实加大现代农业投入，整合涉农资金，集中投向农业基础设施和龙头企业、农民合作社等新型经营主体；加大新型工业投入，把工业投资的重点引向高新技术产业和新型工业，力争工业园区有一批产业项目落地。启动“十三五”重大项目前期工作。着力推进交通枢纽、城市路网、产业园区、社会民生、生态环保、城市功能完善、城市岸线开发、能源结构调整、城市综合防灾、卫星城镇建设等 “十三五” 十大重大项目前期工作。

　　进一步扩大消费。整合各种资源，组织各方力量，加大城市品牌推广和宣传力度。继续抓好旅游、房地产、热带特色农产品“三大促销”活动，创新促销方式，加强网络营销，力争组织大型岛外促销活动达到50场次、规模以上会展活动超过200场次；接待国内外过夜游客突破1120万人次，旅游总收入超过135亿元，增长12%以上；完成商品房销售超过400万平方米。切实推进国家信息消费试点城市建设，加快发展电子商务，培育新的消费增长点。深化“万村千乡市场工程”和“新网工程”建设，扩大农村消费。力争全年社会消费品零售总额超过550亿元。

　　大力推进新型工业发展。继续壮大汽车制造、医药、食品饮料及农副产品加工、机电制造等支柱产业，发展新能源、新材料、电子信息、节能环保等新兴产业， 促进传统产业和优势企业增产扩容，力争全年工业增加值增长10%。推动园区集约集聚发展。按照新型工业向园区集中的既定思路，突出产业主题，引导关联度高的项目向同一园区集中，主打有研发创意、主题鲜明的新兴产业，力争形成1-2个集群式产业、1-2个链条式产业。进一步完善高新区、综保区、桂林洋开发区等园区基础设施配套，推进美安科技新城和狮子岭二期建设，实现产业园区增容扩区。引导和鼓励企业加大投入，开展产学研合作，建立研发中心，加快开发具有自主知识产权的核心产品，全年新增高新技术企业5家。加大财政资金扶持，落实2亿元工业发展专项资金。强化政银企对接，帮助中小企业缓解融资难问题，为各类企业提供20亿元额度担保贷款。

　　加快服务业转型升级。创新旅游发展新业态。主攻商务旅游和休闲旅游，突出发展邮轮游艇、文化旅游、乡村旅游、商务会展、医疗康复、运动休闲等旅游新业态，推动西沙游扩大规模、提升品质。加快冯小刚电影公社、兰桂坊、长影环球100等景点景区建设，加大观澜湖国际旅游度假区国家5A级景区创建力度，推动旅游业提质升级。大力发展文化博览产业。加快骑楼片区改造和五公祠、海瑞文化园等项目建设。多层次打造文化赛事活动，放大万春会、换花节、冼夫人文化节、旅游婚庆节和观澜湖高尔夫世界明星赛等活动品牌效应，促进文化旅游融合。继续加快金融业和总部经济发展。完成兴业银行海口分行、进出口银行海口分行挂牌营业，推动招商证券海口公司、上海仁建集团总部经济落户海口。提升商贸物流中心建设水平。编制完善城市片区商业网点规划，实施国家城市共同配送试点一期项目，加快生生国际、远大商业购物中心、丁村现代美居等商业项目和大印、美安物流园建设，推进林安物流园落地，培育龙头商贸物流企业2-3家。积极调整港航物流优惠政策，推进临空产业园和新海港综合商务区建设。加快房地产结构调整。按照省里“一降一增一保”的要求，重点发展旅游地产、商业地产，降低普通商品住房开发比例，争取用两年时间将商品住房新开工面积比重下降5%以上。

　　（三）围绕农民增收，狠抓“三农”工作

　　加强农村基础设施建设。在农业资源环境保护、水利基础建设、农业科技推广、土地综合整治等方面增加投入，除示范镇外，启动18个农村基础设施项目建设。完成南渡江流域土地整治15万亩，适时启动建设11万亩，确保防洪堤和南面沟泵站主体工程完工。继续推进农村安全饮水、农村公路、农村有线电视等工程建设，解决677户、3048人农村居民安全饮水问题，完成县道改造93.9公里，新改建农村公路56.4公里，新增村级广播站400个、农村有线电视用户5000户。

　　大力发展现代农业。加大对农业企业申报“三品一标”品牌的指导和扶持，着重培育一批农产品地理标志，力争全年新增无公害农产品认定基地1万亩、无公害产品认证10个，新增省著名商标、省名牌产品5个，新建农产品质量安全追溯示范点10个。大力发展设施农业，完成新坡洋1万亩瓜菜基地建设，高标准新建蔬菜大棚5000亩。加快演丰现代农业产业园、海口兰花产业园、文山沉香产业园等项目建设。加快构建现代农业产业化经营体系，开展农民以土地、劳动力、资金、技术等生产要素入股试点，培育一批专业大户、家庭农场等新型农业经营主体，年内新增农民专业合作社30家、家庭农场示范点5家。大力推进农产品加工园区建设，加强对龙头企业的扶持和引导，力争全年新增省级农业龙头企业5-8家。

　　着力促进农民增收。认真实施新一轮农民增收行动计划。培训农村劳动力1万人，促进转移就业，提高劳务性收入占比。结合乡村休闲旅游开发，鼓励发展“农家乐”，开展休闲农业示范基地星级评选。加强文明生态村建设，加大扶贫开发，出台《加强对贫困村财政金融政策扶持方案》，完成145个贫困村的脱贫任务。完善农民小额贷款、农业保险贴息机制，推广农信社“一小通”小额信贷支农模式，扩大农业保险覆盖面。

　　（四）积极稳妥扎实推进新型城镇化，统筹城乡发展

　　启动新型城镇化规划体系编制。落实中央城镇化会议精神，按照省里提出的“科学规划前提、基础设施配套、特色产业支撑、公共服务保障、特色文化包装、绿色田园环保、社会多元投资、打造营销品牌、吸引外来消费、农民当地就业”的海南特色新型城镇化思路，重点围绕“主城区—卫星城—特色小镇—新型农村社区—美丽村庄” 五级城镇化体系，着手研究和编制我市新型城镇化规划，统筹考虑主城区、中心镇和农村新社区建设，因地制宜，做出特色。

　　加快主城区改造升级。继续推进灵山镇、滨涯村、上贤村、夏瑶村、椰岛片区等已开工棚户区（城中村）改造，加大流水坡、白沙坊—博义盐灶、骑楼、府城、红城湖、新海村和演丰镇墟等棚户区（城中村）改造的前期工作，争取年内开工建设。强化有针对性的招商引资，力争今明两年启动的棚户区（城中村）改造项目占总任务的30%以上。加快推进东寨港片区、江东片区、西海岸南片区、金沙湾片区等新区建设。

　　加快统筹城乡示范镇建设。继续推进云龙、演丰示范镇和百个统筹城乡示范项目建设，争取上半年示范镇部分示范项目可看，年内示范镇、示范项目出形象、可推广。推进示范镇试点扩面，适时启动第二批示范镇建设，争取1个示范镇纳入省级新型城镇化试点。按照“一镇一品”、“一镇一业”的思路，加快镇域经济发展，强化镇墟产业支撑，提升就业、卫生、教育等公共服务能力。

　　（五）加强科学管理，提高社会治理水平

　　认真落实省委、省政府 “科学管理年”的统一部署，把管理放在更加突出的位置，从强化制度建设入手，认真研究出台一些新的、实实在在的改革措施，力争初步形成规范有序、运作协调的城市管理和社会治理的长效机制。

　　深化“城市管理年”活动。加强交通整治。以公交都市创建为契机，发展公共交通，启动滨海大道有轨电车1号线、海秀路（BRT）等大容量公交系统项目建设前期工作；提前开展全市车辆总量和流量控制研究；加快秀英港搬迁，安排主城区道路建设工程28个，进一步优化城市路网；继续优化交通组织，编制中心城区路段改造计划，年内启动解放路至海秀东路、海府路至白龙路、南海大道至海垦路、秀英老城区、府城等5个片区的优化改造；综合整治违禁行驶、乱行乱靠、横穿马路等交通违法违规行为，确保交通秩序进一步好转。继续加大违法建筑整治力度。出台《防控和查处违法建筑暂行办法》和《责任追究办法》，抓好违法建筑分类处置，并结合新型城镇化建设，探索拆后土地利用，建立起防违控违长效机制。着力改善城乡环境卫生。深入开展全国文明城市和卫生城市创建工作，抓好病媒防治工作，全面整治卫生死角，严厉处罚城市主次干道占道经营，规范临街道路户外广告设置，开展夜市摊点规范管理试点，创建整洁、有序的城市环境。加强城市综合管理信息平台建设，做到所有在建工地、示范街区广告牌和占道经营等信息全部上图入库，积水点全部实现视频监控，所有渣土车实行GPS跟踪。

　　加强生态环境保护。持续抓好“绿化宝岛”工程，新增造林面积1万亩。加大大气综合整治，重点整治机动车排气污染、建筑施工和裸露地面扬尘、露天烧烤，确保我市大气环境质量一流。持续改善水环境，开展绕城高速以北区域环境面源整治，编制完成中心城区水网动力补水及调度方案，继续实施水环境整治二期工程，加快主城区排污口截流并网改造，推进大同沟、电力村沟、东西湖周边污水管网体系建设。完成白沙门污水处理厂一期升级改造，加快推进垃圾焚烧发电厂扩建，启动颜春岭垃圾填埋场二期建设。严格落实节能减排目标考核责任制，全面完成省里下达的节能减排任务。

　　加强自然资源和公共资源管理。从严管理土地成片开发、沿海沿江岸线、填海造地、林地、海洋等资源，开展岸线、水库、河道、采砂等水资源领域突出问题专项治理。合理调节土地、水、电、气等资源能源价格，充分反映市场供求、资源稀缺程度、生态环境损害成本和修复效益。创新给排水、公共交通、路牌广告、停车场等特许经营权管理，充分运用市场竞争机制来配置，切实保障社会公共利益、公共安全和特许经营者的合法权益。

　　强化社会治理。完善重大决策社会稳定风险评估机制和社会预警体系，提高应急管理水平。重视食品药品安全，对违法违规者实行最严厉的处罚制度和问责制度，确保人民群众“舌尖上的安全”。落实各项安全生产防范措施，加强综合防灾减灾能力建设，坚决遏制重特大事故发生。创新化解矛盾体制机制，改革信访工作制度，运用法治思维和法治方式化解社会矛盾。健全突发事件应急管理机制，深入推进“平安海口”建设，确保社会安全稳定。大力发展社区志愿者服务组织，推动社团组织有序发展。

　　（六）着力保障改善民生，健全基本民生保障体系

　　把就业工作摆到突出位置。实施离校未就业毕业生的“就业促进计划”， 落实创业培训补贴、小额担保贷款及贴息、税费减免等政策，加强对被征地农民、城镇失业人员就业服务和创业培训，扶持创业孵化基地建设，完成城镇新增就业5.5万人，转移农村劳动力就业1万人，确保“零就业”家庭动态清零。

　　全力保持市场物价稳定。切实抓好“菜篮子”工程，分期分批改造现有常年蔬菜基地，高标准建设蔬菜大棚，对农业龙头企业实行“以供定补”的政策资金扶持方式。升级改造南北水果蔬菜批发市场，支持中商海南农产品中心市场建设，做好4个公益性蔬菜批发市场经营管理，改造升级一批农贸市场。继续发挥价格调节基金的作用，落实对困难群体的动态补贴政策。加强市场秩序监管，严厉打击欺行霸市、价格欺诈等现象。

　　提高社会保障水平。抓好失地农民社会保险参保工作，确保新农保、城镇居民养老保险参保率达95%以上，城镇居民医保征缴参保率达98%以上，力争基本社会保险覆盖率指数排在全省前列。积极探索城乡居民养老、医疗保险制度一体化。进一步强化住房保障，完善保障性住房配售机制，完成省政府下达农村危房改造和保障性安居工程建设任务。

　　加快发展社会事业。推进义务教育学校标准化、信息化建设，深入实施薄弱学校改造工程、农村初中校舍工程。加快港湾学校、玉沙学校（二期）、美苑小学、秀华学校、五源河学校新建和市二中改扩建等重点项目建设。积极发展文化事业，完善城乡公共文化服务体系，加快五源河文体中心建设，启动4个区级文化馆、16个村级文化室建设，推动文化馆、图书馆、文化站免费开放。繁荣传统文化，打造特色文化精品。探索城乡医院一体化管理（医疗联合体）服务模式改革，加强农村医疗机构标准化建设，力争创建1—2家省级示范社区卫生服务中心。实施一方是独生子女的夫妇可生育两个孩子的政策，完善计生奖励扶持政策，继续稳定低生育水平，提高出生人口素质。统筹推进经济发展和国防建设，认真做好国防动员、优抚安置、双拥创建工作。深化文明创建，关心支持工会、共青团、工商联、妇女儿童工作，进一步做好外事侨务、民族宗教、民防、气象、档案、邮政、通信、史志、老龄、残疾人等工作，促进社会事业全面发展。

　　各位代表，人民群众的期盼就是政府工作的方向。今年，我们将重点办好八个方面的民生实事。一是实施主城区路网畅通工程，打通民声路通道，优化滨海大道交通组织疏导；启动金鼎路、长兴路等7条道路贯通工程。二是实施乡村通路通邮工程，改建农村公路56.4公里，建设村邮政站141个。三是实施生态环境整治工程，完成8个镇87个行政村890个自然村农村生活垃圾收运；加快金牛湖水环境净化美化工程；继续实施东寨港国家级自然保护区环境综合整治工程。四是实施失地农民保障工程，解决1.78万名被征地农民养老保险参保遗留问题。五是实施社区绿化亮化工程，新建小游园5个，启动万绿园便民休闲道路绿化、照明配套建设；启动金牛岭景区亮灯工程，建设安装金宇西路等16条道路照明设施。六是实施公益性湿地公园建设工程，完成沙坡—白水塘湿地公园一期建设。七是实施城乡教育提升工程，推进桂林洋“大学城”功能完善；加快“麻雀”学校整合，在原市青少年宫新建一所九年制义务教育学校，在市旅职校白龙校区原址改扩建美兰高级实验小学；建成乡镇公办幼儿园4所；新建13所乡村学校少年宫。八是实施城乡安居工程，推进农村危房改造3500户；继续解决房产办证遗留问题。

　　四、加强政府自身建设

　　（一）着力转变政府职能。正确处理市场与政府的关系，把更多精力放在创造良好发展环境、提供优质公共服务、维护社会公平正义上来。进一步简政放权，再下放30项市级行政审批服务事项，网上审批覆盖率达60%以上，行政审批提速15%。承接好省级下放行政审批事项，深化强区扩权，完善政府购买公共服务机制。推进政府机构改革，统筹抓好职能调整。完善绩效评价体系，创新监管体制机制，建立健全目标考核、规范运行、工作落实、责任追究等机制，确保各项工作能落实、快落实。

　　（二）着力推进依法行政。自觉接受人大法律监督、政协民主监督，认真办理人大议案、代表建议和政协提案。注重发挥工会、共青团、妇联等人民团体桥梁纽带作用，广泛听取民主党派、工商联、无党派人士意见。重视新闻媒体监督和公众舆论监督，拓展政府与群众的互动交流平台。健全政务、厂务、村务公开等制度，不断扩大基层民主。坚持重大事项集体讨论决定，完善公众参与、专家论证和政府决策相结合的机制，健全社会听证制度。加强网上政务服务和电子监察平台建设与应用，提高政府工作透明度。普遍建立法律顾问制度，完善规范性文件、重大决策合法性审查机制。强化行政执法监督，严格实行行政执法责任制和过错追究制，规范行政执法自由裁量权。开展相对集中行政复议权试点。

　　（三）着力加强政风建设。坚决反对“四风”，认真开展党的群众路线教育实践活动，深入基层、深入企业、深入群众，倾听群众呼声、集中群众智慧，着力解决群众反映的突出问题。坚持勤俭办一切事业，严格控制“三公”消费和一般性开支，严格限制楼堂馆所建设，规范公务用车管理，在政府自觉过“紧日子”中让人民过上好日子。全面落实党风廉政建设“一岗双责”，严格执行领导干部个人有关事项报告制度，用制度管权、管事、管人。强化行政监察和审计监督。继续整治庸懒散奢贪，严肃查处吃拿卡要等行为，做到有令必行、有禁必止。深入开展纠风和专项治理，坚决纠正部门利益固化和损害群众利益的不正之风。

　　各位代表！“真抓才能攻坚克难，实干才能梦想成真”。全面完成今年各项经济社会发展任务，需要全市上下同心协力、埋头苦干。让我们更加紧密团结在以习近平同志为核心的党中央周围，在省委、省政府和市委的坚强领导下，改革创新，扎实工作，以“一天也不耽误”的精神，为建设海南“首善之城”和“四宜三养”最精最美省会城市不懈奋斗！
